# Effectiveness and Safety of Electroacupuncture for Depression: A Systematic Review and Meta-Analysis

**DOI:** 10.1155/2022/4414113

**Published:** 2022-08-18

**Authors:** Zhuo Zhou, Guixing Xu, Liuyang Huang, Hao Tian, Fengyuan Huang, Yilin Liu, Mingsheng Sun, Fanrong Liang

**Affiliations:** The Acupuncture and Tuina School, Chengdu University of Traditional Chinese Medicine, Chengdu, Sichuan, China

## Abstract

**Purpose:**

The purpose of this systematic review and meta-analysis was to comprehensively evaluate the efficacy and safety of electroacupuncture as an effective adjunctive therapy for patients with depression.

**Methods:**

Randomized controlled trials (RCTs) about the treatment of depression by electroacupuncture therapy from inception to September 2021 were searched and collected in eight databases. HAMD, SDS, and Adverse Reactions were used as outcome indicators. The quality of relevant articles was evaluated using the Cochrane Collaboration's risk of bias tool. The quality of evidence for each outcome was assessed through the Grading of Recommendations Assessment and the Development and Evaluation approach. Stata 15.0 software was used for data analysis.

**Results:**

A total of 16 depression-related RCTs were included in this meta-analysis. For the main outcome, electroacupuncture significantly reduced HAMD scores (I^2^:0.0%, SMD: −2.28% (95% CI−3.16 to −1.39)), and the quality of evidence was moderate. The improvement effect of electroacupuncture plus antidepressants was better than that of western drugs in patients with depression (I^2^:26.2%, SMD: −1.18% (95% CI−1.42 to −0.94)), and the quality of evidence was moderate. Electroacupuncture significantly reduced HAMD scores without significant heterogeneity (I^2^:0.0%, SMD: −3.76% (95%CI−5.78−1.73)). Studies with very low quality of evidence found that electroacupuncture was as effective as antidepressants in reducing SDS scores (I^2^:36.4%, WMD: −1.15% (95%CI−2.93–0.63)), and electroacupuncture was found to be more effective than sham electroacupuncture stimulation as well. Moderate quality evidence showed no statistical difference between electroacupuncture plus antidepressants/electroacupuncture and antidepressants (I^2^:0%, RR:1.05% (95%CI 0.73 to 1.53)).

**Conclusions:**

Our meta-analysis shows that electroacupuncture reduces HAMD scores. It is suggested to use electroacupuncture plus antidepressants to improve the curative effect and effectively reduce drug side effects.

## 1. Introduction

Depression is a common emotional disorder syndrome, mainly characterized by significant and persistent low spirits. Loss of interest, attention difficulties, feelings of guilt, loss of appetite, even suicidal thoughts, and other cognitive, behavioral, and social dysfunction are the main clinical manifestations of depression [1]. A persistent state of depression can cause serious harm to individuals and society. According to the World Health Organization, more than 300 million people are living with depression all around the world. A study showed that major depression is the most common mood disorder in China, with a lifetime prevalence of 3.4% and a 12-month prevalence of 2.1% [2]. At the same time, a meta-analysis has shown that depression treatment brings tremendous socioeconomic pressure [3], and depression is also ranked as the single largest contributor to nonfatal health loss [4].

The treatment of depression mainly includes psychotherapy, complementary and alternative medicine (CAM), exercise, and pharmacotherapy. However, due to the accelerated pace of modern life, drugs are often used as the treatment of choice. The second-generation antidepressants (SGAs) (selective serotonin reuptake inhibitors, serotonin norepinephrine reuptake inhibitors, and selective serotonin norepinephrine reuptake inhibitors) are the main drugs used to alleviate depression in clinical practice [5]. But studies [6] have shown that the drug has serious adverse reactions, even neuroleptic malignant syndrome (NMS), and a lack of safety for pregnant women and the elderly [7]. Therefore, it is urgent to find an effective and reliable alternative therapy to treat depression.

Acupuncture, as an intervention of traditional Chinese medicine, is widely used to treat neuropsychiatric diseases [8]. Studies have shown that acupuncture can regulate the limbic‐paralimbic‐neocortical network (LPNN), thus helping to improve the life and sleep quality of patients with depression [9]. Also, it has been recommended as a complementary alternative therapy in the United States [10]. Electroacupuncture (EA), a part of traditional acupuncture, is a combination of acupuncture and electrical stimulation that objectively controls the amount of stimulation. Through research, Sun et al. [11] proved that electroacupuncture is as effective as anti-depressants in the treatment of depression. Although some studies have provided partial evidence for EA in the treatment of depression [12], there is still a lack of high-quality RCTs to confirm the effectiveness and safety of EA for depression on the whole. Therefore, we included clinical studies related to EA treatment of depression with a Jadad score greater than or equal to 4. The primary outcome was the HAMD scale, and the secondary outcome was the SDS scale. At the same time, the safety of EA therapy in patients with depression is evaluated to report a more advanced objective validation of available data.

## 2. Materials and Methods

### 2.1. Study Registration

The SRs-MAs research was conducted in accordance with the Preferred Reporting Items for Systematic Reviews and Meta-Analyses (PRISMA) statement. It was registered at INPLASY's International Prospective Register of Systematic Reviews (INPLASY202210068).

### 2.2. Eligibility Criteria

#### 2.2.1. Types of Study

This study included all RCTs that have published Chinese or English articles about electroacupuncture for depression. At the same time, nonrandomized controlled trials, quasi-randomized controlled trials, conference papers, retrospective studies, medical record reports, protocols, reviews, article abstracts, and meta-analyses were excluded.

#### 2.2.2. Types of Participants

All patients included in this study were diagnosed with depression according to [10] (ICD-10), the Diagnostic and Statistical Manual [1] (DSM-V, May 2013), or the Criteria for Classification and Diagnosis of Mental Diseases approved by the Chinese Psychiatric Science Committee [13].

#### 2.2.3. Criteria for Intervention

Studies of EA for depression were included. Whether EA was used alone as an intervention or in combination with other treatments (western medicine, moxibustion, transcranial electrical stimulation, and so on) can be included.

#### 2.2.4. Criteria for Comparison

The control group was either treated with traditional western medicine, placebo, sham acupuncture, EA, or waited for treatment (with no intervention during the study). Psychological therapies (behavioral therapy, cognitive behavioral therapy, psychological counseling, etc.) were excluded from the intervention of the control group because of their strong subjectivity.

#### 2.2.5. Outcome

The Hamilton Depression Rating Scale for Depression (HAMD17/24) was considered as the primary outcome index of this study, while the self-rating depression scale (SDS) and adverse reactions that occurred during treatment were regarded as secondary outcome indexes. Adverse reactions and follow-up data were statistically analyzed to evaluate the safety of electroacupuncture for depression treatment.

### 2.3. Search Strategy

The following eight databases will be searched from inception to 10th September 2021: PubMed, Embase, Cochrane Library, Web of Science, China National Knowledge Infrastructure (CNKI), Chinese Biomedical Literature Database (CBM), VIP Database, and WanFang Database. We followed key search terms and their potential combinations on PubMed: (1) clinical condition: depression, depressive disorders, depress, Journal Pre-proof “affective disorder, affective symptoms; (2) acupuncture terms: electroacupuncture, Electric acupuncture, EA, Electroacupuncture therapy, Electroacupuncture therapy, Electric acupuncture therapy; (3) study type: randomized controlled trial. We used “and” and “or” to connect the search terms. Search strategies are shown in Table 1.

### 2.4. Study Selection and Data Extraction

In this study, two reviewers (ZZ and XGX) searched the above databases in no particular language. After preliminary screening according to the titles and abstracts of the articles, duplicate references were deleted using Endnote. After full-text reading, the remaining articles that could be included in this study were identified, and then information extraction was carried out. Two reviewers (ZZ and XGX) independently extracted data from the included trials, mainly including the data extraction table name, author, name of the magazine, published literature, diagnostic criteria, number, time of intervention, treatment, and outcome indicators, adverse events, and other aspects. These data were recorded by Office 2019. If the information is incomplete, we will contact the authors by phone or e-mail to acquire more information. When disagreements arise in the research, they are resolved through discussion or consultation with a third reviewer (LFR) until consensus is reached.

### 2.5. Risk of Bias and Quality Assessment

Two reviewers (HLY and TH) independently assessed the risk of bias in the final included studies using the risk of bias assessment tool developed by the Cochrane Collaboration [14]. This standard included six items of selection bias, performance bias, detection bias, attrition bias, reporting bias, and other biases, and each standard was divided into “low” bias risk, “high” bias risk, and “unclear” bias risk. Two independent researchers (HFY and LYL) evaluated the legal quality by the Jadad scale [15]. Any ambiguity in the evaluation process was resolved by LFR. Only high-quality studies with Jadad scores greater than or equal to 4 were included in this study.

After the Cochrane Collaboration's Bias Risk Tool and Jadad score were completed, we graded the quality of evidence for each study. The Grading of Recommendations Assessment, Development, and Evaluations (GRADE) system was used to evaluate the evidence quality of the outcomes [16]. The evaluation of the level of evidence included risk of bias, inconsistency, indirectness, imprecision, and publication bias, and it was divided into four grades: high, moderate, low, and very low.

### 2.6. Statistical Analysis

Statistical analyses were performed using STATA 15.0 software in our study. After the inclusion information was sorted out, groups were divided according to the type of intervention and control method. If the RCTs set up three matched control groups, they were split and compared in pairs. For the main outcome indicators—HAMD-17/24, we converted the corresponding scores of HAMD-17 into the scores of phase HAMD-24. Meanwhile, we unified the units of each result of different trials according to the international system of units and then imported clinical data into STATA 15.0.

Random-effects models were used to analyze dichotomous and continuous data for risk ratio (RR), standard mean difference (SMD), and 95% confidence intervals (95%CI). Q statistics were used to investigate the heterogeneity between studies, *P* values <0.10 were considered an indicator of significant heterogeneity. When the heterogeneity test I^2^ < 75%, the random effects model was used for data synthesis. If there was considerable heterogeneity in the experiment (I^2^ > 75% or*P* < 0.10), each trial was removed from the population analysis. At the same time, sensitivity analysis was performed to identify studies that significantly affected the population effect. When we included enough trials, the funnel plot was visually examined, and Egger or Begg tests were used to quantitatively evaluate publication bias. The subgroup analysis or meta-regression was not conducted due to a few heterogeneities.

## 3. Results

### 3.1. Results on Literature Search and Selection

**Figure 1:**
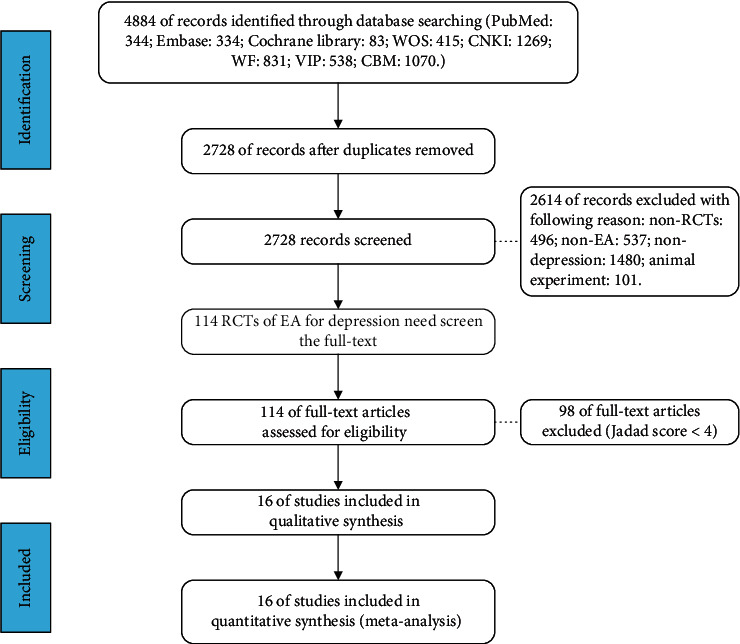
Flowchart of study selection.

We obtained 4884 records from the eight databases through the retrieval strategy in advance and eliminated 2728 duplicate records before preliminary screening. After reading the titles and abstracts, 114 eligible studies were left. Jadad scores were performed on the literature, and 98 trials with Jadad scores of less than 4 points were excluded.  Table 2 shows the Jadad score of the included trial (Jadad ≥4). A total of 16 RCTs met the inclusion criteria. Study selection flow chart is shown in Figure 1.

### 3.2. Characteristics of Included Reviews

Our study included 16 [11, 17–31] RCTs of electroacupuncture/electroacupuncture plus antidepressant drugs versus sham-electroacupuncture/antidepressants. There are two control groups for Sun H et al. [11, 20, 25], 8 trials [11, 19, 21–24, 26, 31] used electroacupuncture alone as an intervention, and 8 studies [17, 18, 20, 27–31] used acupuncture plus antidepressants. A total of 1428 participants were enrolled in the trial with a sample size range of 316∼40 and a mean age of 40 (range 30∼48). At the baseline, the mean HAMD-24 score was 37.04 (range23.8–47.36). The main characteristics and the specific interventions of each identified study are presented in Table 3.

### 3.3. Risk of Bias Assessment

All of the 16 studies [11, 17–31] mentioned the random sequence generation methods, including computer program random sequencing, random number tables, or random number generators, and were assessed as having a low risk of bias. Among the allocation concealment methods, 8 studies [11, 17, 23, 24, 26–28, 31] were carried out using the sealed envelope method, the random list method, the random allocation method, or the coin method to determine single and even grouping, with a low risk of bias, while the other 8 trials [18–22, 25, 29, 30] were unclear because they did not mention or describe any method of allocation concealment. Due to the active manipulation of EA, most clinical studies could not be completely blinded. About the blinding of participants and investigators, 7 studies [11, 22, 23, 26, 27, 29, 30] were high-risk studies, 5 RCTs [17, 20, 21, 28, 31] were unclear, and only 4 [18, 24–26] of blindness were evaluated as low risk. 4 RCTs [18, 24–26] reported in detail the outcome assessment of blindness and were rated as low risk. In terms of data integrity, all 16 studies have been described in detail, so we believe that all studies have a low risk of bias. In the 9 studies [17, 18, 20, 21, 24–28, 30, 31] we included all expected outcomes reported, including adverse events, and were assessed as low risk of bias. The remaining studies either did not document the protocol or did not mention adverse events, so the risks were not clear. Other sources of bias, 2 [11, 27] high-risk, 4 [17, 18, 20, 31] low-risk, others unclear.  Table 4 presents the risk of bias in each trial.

### 3.4. Effectiveness of EA

#### 3.4.1. HAMD-24

**Figure 2:**
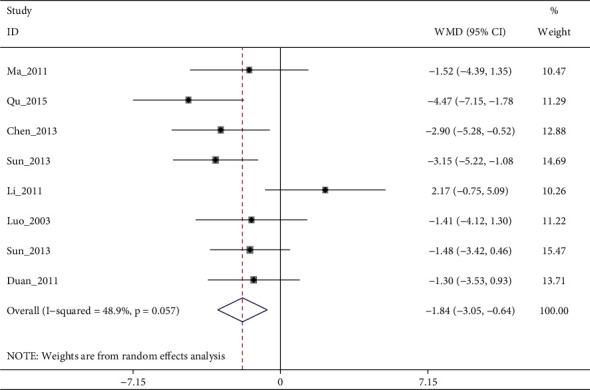
Forest diagram of HAMD-24 for electroacupuncture vs. antidepressants.

**Figure 3:**
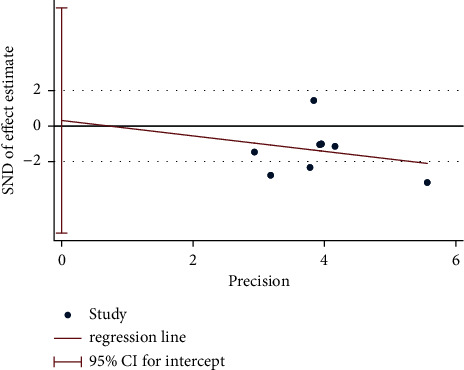
Egger test of HAMD for electroacupuncture vs. antidepressants.

**Figure 4:**
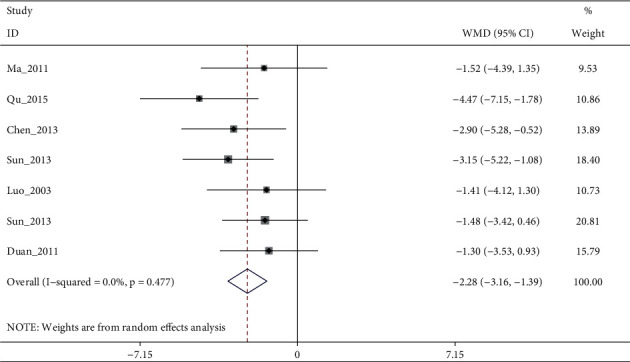
Forest diagram of HAMD for electroacupuncture vs antidepressants removed Li_2011.

**Figure 5:**
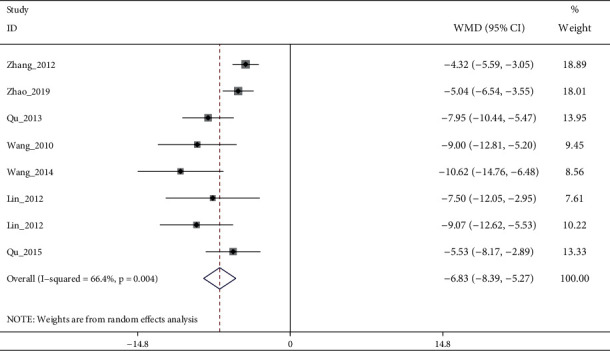
Forest diagram of HAMD-24 for electroacupuncture plus antidepressants vs antidepressants.

**Figure 6:**
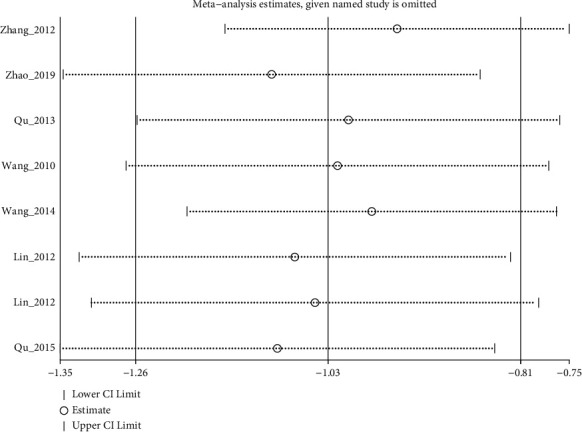
Sensitivity analyses of electroacupuncture plus antidepressants vs. antidepressants for HAMD-24.

**Figure 7:**
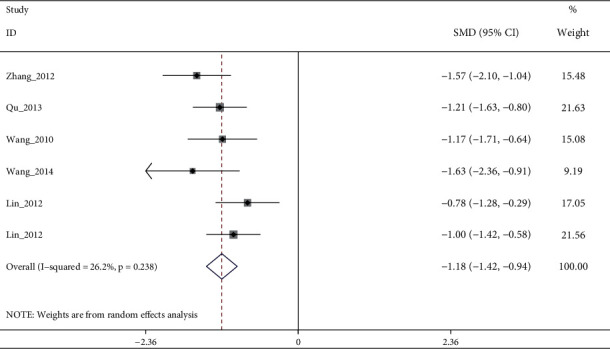
Forest diagram of HAMD for electroacupuncture plus anti-depressants vs. antidepressants removed Zhao_2019.

A total of 15 RCTs (except [19]) investigated the effect of electroacupuncture on HAMD scores in our meta. Meta-analysis of 7 RCTs (Sun_2013 [11] had two control groups) involving 8 trials (506 participants) revealed significant differences in HAMD score reduction between electroacupuncture and antidepressants (SMD: −1.84 (95% CI −3.05 to −0.64), *I*^2^:48.9%, Figure 2), and no significant publication bias (Figure 3). Heterogeneity disappeared after Li_2011 [23]was removed (I2:0.0%, SMD: −2.28% (95% CI−3.16 to −1.39), with moderate quality evidence (Figure 4 and Table 5). Pooled 8 studies found that electroacupuncture plus antidepressants was more effective in improving the patients' depression (SMD: −6.83% (95% CI −8.39 to −5.27), I2:66.4%, Figure 5), compared with antidepressants. Heterogeneity was significantly reduced after Zhao_2019 [17] was removed and evidence quality was moderate (I2:26.2%, SMD: −1.18% (95% CI−1.42 to −0.94), Figure 6, Figure 7, and Table 5). There were only 2 studies [25, 26]that compared electroacupuncture with sham electroacupuncture, and the quality of evidence was moderate (I2: 0.0%, SMD: −3.76% (95% CI−5.78 to −1.73)).

**Figure 8:**
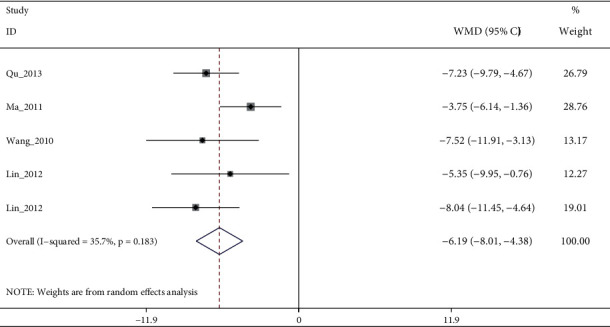
Forest diagram of HAMD for electroacupuncture vs. antidepressants in follow-up.

In follow-up, a meta-analysis of 6 studies with moderate quality evidence showed that electroacupuncture plus antidepressants therapy was more effective than antidepressants therapy alone (I2: 35.7%, WMD: −6.19% (95% CI−8.01 to −4.38), Figure 8).

#### 3.4.2. SDS

**Figure 9:**
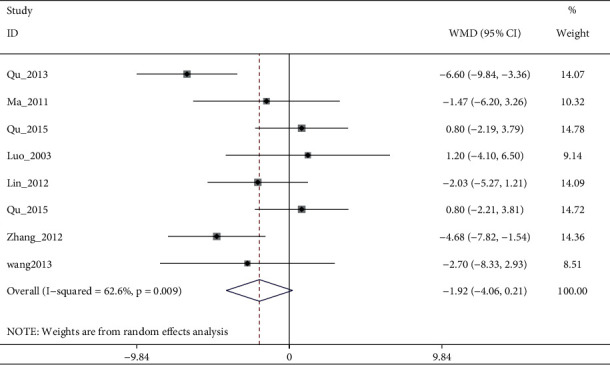
Forest diagram of SDS for electroacupuncture vs. antidepressants.

**Figure 10:**
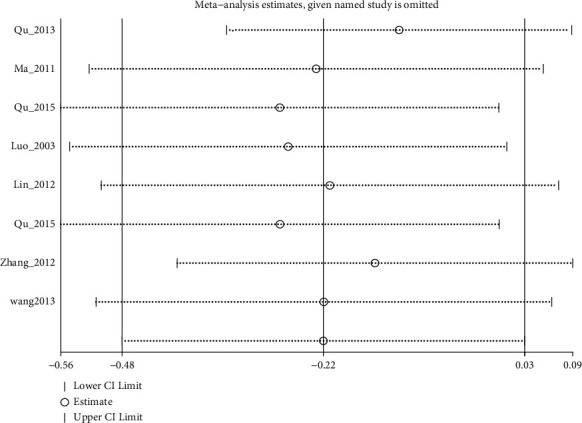
Sensitivity analyses of electroacupuncture vs. antidepressants for SDS.

**Figure 11:**
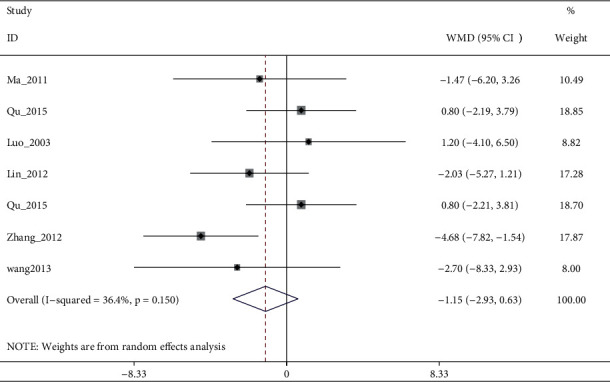
Forest diagram of SDS for electroacupuncture vs. antidepressants removed Qu_2013.

SDS scores were mentioned in the results of 9 trials. Pooled 7 studies found that electroacupuncture alone or plus antidepressants and antidepressants had no significant differences in reducing SDS (I2:62.6%, WMD: −1.92% (95% CI −4.06 to 0.21), see Figure 9). Although sensitivity analysis reduced heterogeneity (I2:36.4%, WMD: −1.15% (95% CI −2.93 to 0.63), Figure 10, and Figure 11), the quality of evidence was very low (Table 5). The remaining two studies [8, 25] suggested that electroacupuncture was more effective than sham electroacupuncture (WMD: −5.39 (95% CI: −8.82 to −1.97), I2 = 0%), and the quality of evidence was high.

In follow-up, electroacupuncture may be more effective than antidepressants (I^2^: 89.1%, WMD: −6.19 (95% CI −10.87 to −4.67)) with moderate quality of evidence.

### 3.5. Safety of EA

**Figure 12:**
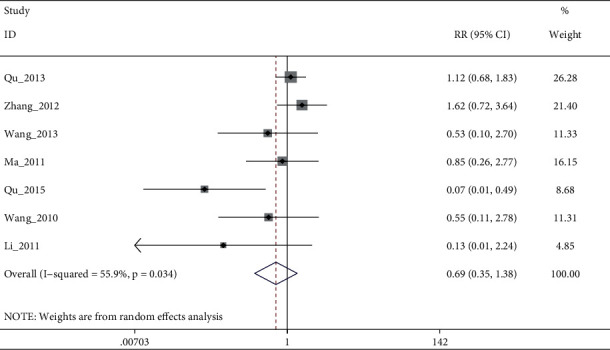
Forest diagram of adverse events for electroacupuncture vs. antidepressants.

**Figure 13:**
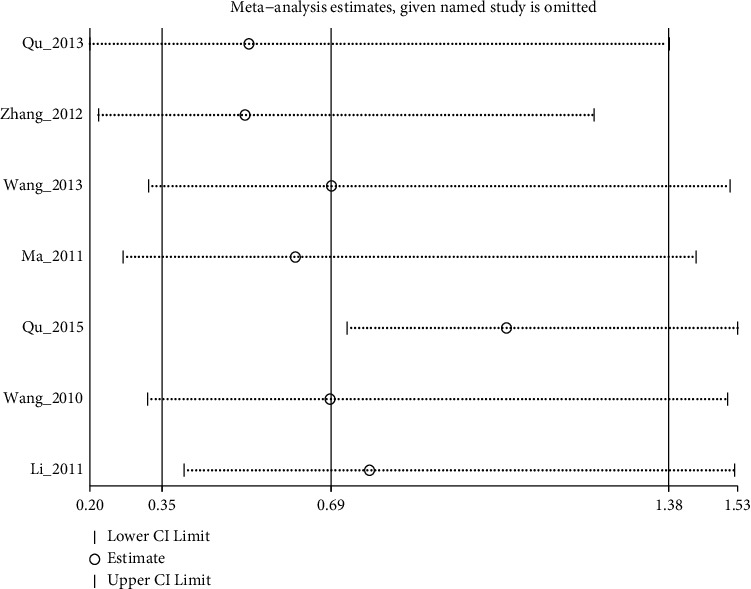
Sensitivity analyses of adverse events for electroacupuncture vs. antidepressants.

**Figure 14:**
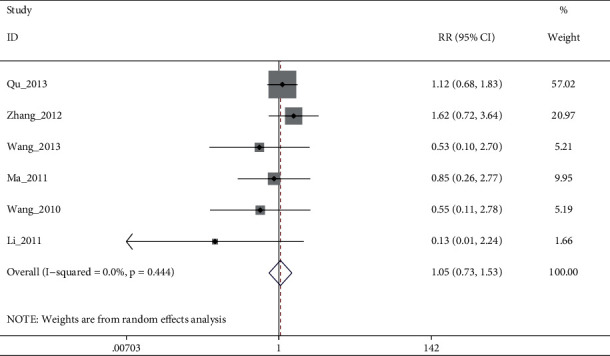
Forest diagram of adverse events for electroacupuncture vs. antidepressants.

Pooled 7 studies showed no statistical difference between electroacupuncture plus antidepressants/electroacupuncture and antidepressants, with moderate quality evidence (I^2^: 55.9%, RR: 0.69% (95% CI 0.73 to 1.38), see Figure 12). After sensitivity analysis and the elimination of Qu_2015, heterogeneity disappeared, but there was still no significant difference. (I^2^: 0%, RR: 1.05% (95% CI 0.73 to 1.53), see Figure 13 and Figure 14)

## 4. Discussion

Our meta-analysis included 16 high-quality RCTs with EA as the primary intervention, involving 1428 patients with depression, and we found that EA is promising as an alternative treatment for depression. The significant effect of EA on depressed patients was not a placebo effect, and EA/EA plus antidepressants therapy can significantly reduce the HAMD scores and SDS scores compared with drugs. In addition, as an adjunctive therapy, EA can not only significantly improve the clinical therapeutic effect but also effectively reduce the incidence of adverse drug reactions.

The pathogenesis of depression is mainly related to the insufficiency of neurotransmitters such as 5-hydroxytryptamine (5-HT) and noradrenalin (NE) in the brain. Besides, Gammaaminobutyric acid (GABA), brain-derived neurotrophic factor (BDNF), and dopamine (DA) also participate in the pathogenetic process of depression [32, 33]. There is growing evidence that EA has benefits in treating depression. Study [34] has suggested that EA can improve the synaptic plasticity of the prefrontal cortex by upregulating GluR1 and NR2B expression levels, thus contributing to antidepressant effects. Li et al. [35] found that EA may improve the depression behavior of rats by upregulating p11mRNA expression in the raphe nucleus and promoting 5-htr4 expression in the plasma membrane of glutamate neurons. Although similar meta-analyses have been conducted in the past to prove the effectiveness of acupuncture on depression [36–38], compared with Zhang et al. [36] and Wang et al. [37], we only included studies with a Jadad score ≥4 to ensure the high quality of research results. At the same time, our study excluded the influence of other treatment methods on the study and only compared the difference between EA therapy/drugs/sham EA therapy, and excluded the comforting effect of EA. More critically, in addition to focusing on the adverse effects of EA for depression, we also explored the follow-up period to definitively determine the long-term efficacy of EA for depression.

Due to the characteristics of EA operation, the deficiency of blindness is the biggest reason for the deviation. In addition, the included study section did not report in detail the calculation of its sample size. Differences in sample size and course of the disease, the selection of acupoints, and EA frequency of studies increase the heterogeneity, which all reduce the level of evidence. There are some other limitations. All the studies were conducted in China, where acupuncture has a high acceptance as a traditional form of treatment, which may be another source of bias. Besides, this study only regarded electroacupuncture as the only intervention of TCM and neglected to explore the combined use of other treatment methods for better efficacy.

Acupuncture is not the only treatment for depression, but our study demonstrates that EA can significantly improve symptoms of depression. It also reduces the side effects of medication as a safe treatment. However, depression is a chronic and relapsing disease associated with mood, and a large-sample size and long-term follow-up studies are still needed in future studies. It is necessary for us to explore the optimal treatment frequency and acupoint selection of EA therapy to understand its long-term effects for better clinical application. Thus, EA treatment of depression has greater clinical application space. We hope that more high-quality, large-sample studies will confirm our findings in the future.

## 5. Conclusions

In summary, despite the above limitations, the high-quality RCTs we included still suggested that electroacupuncture could reduce HAMD score, and we also suggested that electroacupuncture plus antidepressants should be used to enhance efficacy and effectively reduce drug side effects.

## Figures and Tables

**Table 1:** Search strategy for Cochrane library database.

#1	Randomized controlled trial [All text]
#2	Controlled clinical trial [All text]
#3	Randomized [All text]
#4	Randomized [All text]
#5	Placebo [All text]
#6	Randomly [All text]
#7	Trial [All text]
#8	Groups [All text]
#9	Or/#1– #8
#10	Depression [Title/Abstract/Keyword]
#11	Depressive disorder [Title/Abstract/Keyword]
#12	#10 or #11
#13	Electroacupuncture [Title/Abstract/Keyword]
#14	Electric acupuncture [Title/Abstract/Keyword]
#15	EA [Title/Abstract/Keyword]
#16	Electroacupuncture therapy [Title/Abstract/Keyword]
#17	Electroacupuncture therapy [Title/Abstract/Keyword]
#18	Electric acupuncture therapy [Title/Abstract/Keyword]
#19	Or/#13-#19cWarm needle [Title/Abstract/Keyword]
#20	#9 and #12 and #19

This search strategy was modified to be suitable for other electronic databases.

**Table 2:** Jadad score in included studies.

Study ID	Random sequence generation	Allocation concealment	Blinding	Withdrawals and lost visits	Total
Ma_2011	2	2	2	1	7
wang_2014	2	2	2	1	7
Qu_2015	2	2	1	1	6
Wang_2010	2	2	1	1	6
Zhao_2019	2	2	1	1	6
Luo_2003	1	1	2	1	5
Li_2011	2	2	0	1	5
Wang_2014	2	2	0	1	5
Duan_2011	2	1	1	1	5
Qu_2013	2	1	1	1	5
Sun_2013	2	2	0	1	5
Zhang_2012	2	1	2	0	5
Lin_2012	2	1	0	1	4
Lin_2012	2	1	0	1	4
Chen_2013	2	1	0	1	4
Wang_2013	2	1	0	1	4

**Table 3:** The characteristics of each identified study.

		Experimental group		Control group		Control group		Course	Sample sizes			Sex			Age			Course of disease			HAMD-24			Adverse reactions		
Study ID	Diagnostic criteria	Therapeutics	Times	Therapeutics	Times	Therapeutics	Times		Experimental	Control	Control	Experimental	Control	Control	Experimental	Control	Control	Experimental	Control	Control	Experimental	Control	Control	Experimental	Control	Control
Zhao_2019	ICD-10	EA + D	18	SSRIs	NA	NA	NA	6Weeks	160	156	NA	52	57	NA	41.18 ± 12.00	41.76 ± 12.85	NA	10.77 ± 15.32	9.52 ± 13.85	NA	45.36 ± 5.53	44.32 ± 5.42	NA	NA	NA	NA
Duan_2011	ICD-10	EA	36	Fluosefine	NA	NA	NA	6Weeks	36	34	NA	13	13	NA	35 ± 8	35 ± 8	NA	7.2 ± 2.4	7.2 ± 2.4	NA	23.8 ± 4	25.1 ± 3.7	NA	NA	NA	NA
Qu_2013	ICD-10	EA + D	18	Paroxetine	NA	NA	NA	6Weeks	58	48	NA	33	19	NA	33.2 ± 9.0	34.4 ± 10.8	NA	19.8 ± 22.4	20.8 ± 26.1	NA	45.19 ± 6.99	42.12 ± 4.03	NA	27	19	NA
Sun_2013	DSM-V	EA	30	EA	30Times	Fluosefine	NA	6Weeks	20	16	25	8	3	3	43.10 ± 13.86	42.56 ± 10.70	40.72 ± 12.80	24.84 ± 8.66	38.4 ± 17.6	28.2 ± 14.9	23.80 ± 2.93	22.81 ± 3.25	23.32 ± 3.49	NA	NA	NA
Zhang_2013	DSM-V	EA + D	9	Fluoxetine	9Times	NA	NA	3Weeks	38	35	NA	11	1	NA	46.36 ± 9.9	48.26 ± 9.8	NA	7.96 ± 8.0	7.36 ± 7.1	NA	43.2 ± 5.11	41.76 ± 4.48	NA	14	7	NA
Chen_2013	CCMD-3	EA	28	Paroxetine	NA	NA	NA	4Weeks	30	30	NA	10	11	NA	43 ± 3	45 ± 4	NA	48 ± 2.42	50.4 ± 2.77	NA	28.17 ± 2.17	28.10 ± 1.18	NA	NA	NA	NA
Wang_2013	DSM-V	EA	72	Paroxetine	NA	NA	NA	24Weeks	30	30	NA	7	8	NA	48.1 ± 13.4	47.1 ± 10.6	NA	9.45 ± 9.24	15.75 ± 21.3	NA	NA	NA	NA	2	4	NA
Ma_2011	ICD-10	EA	18	Paroxetine	NA	NA	NA	6Weeks	28	35	NA	18	20	NA	45.52 ± 13.64	38.30 ± 14.71	NA	3.5 ± 2.69	3.89 ± 3.56	NA	25.57 ± 4. 83	26.29 ± 6.01	NA	4	6	NA
Wang_2014	ICD-10	EA	120	SEA	120Times	NA	NA	12 Weeks	32	32	NA	9	11	NA	43.5 ± 2.61	43.8 ± 2.16	NA	NA	NA	NA	27.56 ± 1.08	26.69 ± 0.67	NA	NA	NA	NA
Qu_2015	ICD-10	EA	18	Paroxetine	NA	NA	NA	6Weeks	64	65	NA	23	31	NA	36.58 ± 10.90	35.58 ± 10.62	NA	10.47 ± 23.25	12.15 ± 1691	NA	36.75 ± 3.99	38.99 ± 4.8	NA	1	19	3
Wang_2010	ICD-10	EA + D	18	Paroxetine	6Weeks	NA	NA	6Weeks	31	32	NA	16	14	NA	32.26 ± 8.73	36.22 ± 11.07	NA	17.58 ± 20.45	19.06 ± 23.78	NA	46.42 ± 6.82	42.64 ± 5.96	NA	2	4	NA
Li_2011	CCMD-3	EA	30	SSRIs	NA	NA	NA	6Weeks	30	30	NA	11	10	NA	40.33 ± 11.61	38.87 ± 12.20	NA	6.37 ± 2.41	6.47 ± 3.33	NA	34.07 ± 4.27	35.07 ± 3.52	NA	0	4	NA
Luo_2003	DSM-V	EA	30	Fluosefine	NA	SEA	30Times	6Weeks	31	32	32	NA	NA	NA	30 ± 11	34 ± 13	32 ± 12	NA	NA	NA	22.42 ± 2.93	22.16 ± 2.16	22.84 ± 3.47	NA	NA	NA
Wang_2014	ICD-10	EA + D	18	Paroxetine	NA	NA	NA	6Weeks	23	17	NA	3	6	NA	47 ± 11	48 ± 9	NA	NA	NA	NA	47.36 ± 5.28	45.98 ± 5.53	NA	NA	NA	NA
Lin_2012	ICD-10	EA + D	18	Paroxetine	NA	NA	NA	6Weeks	34	34	NA	17	14	NA	31.62 ± 8.9	35.21 ± 11.49	NA	NA	NA	NA	47.56 ± 7.35	42.52 ± 5.89	NA	NA	NA	NA
Lin_2012	CCMD-3	EA + D	18	SSRIs	NA	NA	NA	6Weeks	46	54	NA	14	15	NA	47 ± 8	38 ± 10	NA	4.72 ± 2.4	4.48 ± 2.7	NA	45.26 ± 7.42	42.23 ± 5.82	NA	NA	NA	NA

*Note.* EA: electroacupuncture; EA+D: electroacupuncture plus antidepressant drugs; SEA: Sham electroacupuncture. Note. CCMD-2/3 = the third edition of Chinese classification of mental disorders; ICD-10/11 = the International statistical classification of diseases and related health problems; DSM-II/III/III-R/IV/IV/V = the diagnostic and statistical manual of disorders; NA = not applicable.

**Table 4:** Risk of bias in included studies.

Study ID	Random sequence generation	Allocation concealment	Blinding of participants and investigators	Blinding of outcome assessment	Incomplete data	Selective reporting	Other bias
Zhao_2019	Low	Low	Unclear	Unclear	Low	Low	Low
Duan_2011	Low	Unclear	Unclear	Unclear	Low	Low	Unclear
Qu_2013	Low	Unclear	Unclear	Unclear	Low	Low	Low
Sun_2013	Low	Low	High	High	Low	Unclear	High
Zhang_2012	Low	Unclear	Low	Low	Low	Low	Low
Chen_2013	Low	Unclear	High	High	Low	Unclear	Unclear
Wang_2013	Low	Unclear	High	High	Low	Unclear	Unclear
Ma_2011	Low	Low	Low	Low	Low	Low	Unclear
Wang_2014	Low	Low	Low	Low	Low	Low	Unclear
Qu_2015	Low	Low	Unclear	Unclear	Low	Low	Low
Wang_2010	Low	Low	Unclear	Unclear	Low	Unclear	Unclear
Li_2011	Low	Low	High	Unclear	Low	Unclear	Unclear
Luo_2003	Low	Unclear	Low	Low	Low	Low	Unclear
Wang_2014	Low	Low	High	High	Low	Unclear	High
Lin_2012	Low	Unclear	High	High	Low	Low	Unclear
Lin_2012	Low	Unclear	High	High	Low	Unclear	Unclear

**Table 5:** GRADE quality assessment of evidence.

Intervention	Outcome indicators	Risk of bias	Inconsistency	Indirectness	Imprecision	Publication bias	Large effect	Dose-response	All plausible confounding	Quality of evidence
Electroacupuncture versus sham- electroacupuncture	HAMD-24	−1^a^	0	0	0	0	0	0	0	Moderate
	SDS	0	0	0	−1^c^	0	0	0	0	Moderate
	Side effect	0	0	0	0	0	0	0	0	High

Electroacupuncture versus antidepressants	HAMD-24	−1^a^	0	0	0	0	0	0	0	Moderate
	SDS	−1^a^	−1^b^	0	−1^c^	0	0	0	0	Very low
	Side effect	0	−1^b^	0	0	0	0	0	0	Moderate

Electroacupuncture plus antidepressants versus antidepressants	HAMD-24	−1^a^	−1^b^	0	0	0	0	0	0	Low
	SDS	−1^a^	−1^b^	0	0	0	0	0	0	Low
	HAMD-24 follow-up	−1^a^	0	0	0	0	0	0	0	Moderate
	Side effect	0	−1^b^	0	0	0	0	0	0	Moderate

*Note.* GRADE, the grading of recommendations assessment, development and evaluation; HAMD-24, 24-item hamilton rating scale for depression; SDS, zung self-rating depression scale; ^a^included studies have risk of bias; ^b^heterogeneity of meta-analysis; ^c^small sample size; ^d^potential publication bias.

## Data Availability

The data analyzed in this meta-analysis and systematic review are derived from published articles and can be retrieved from the previous eight databases.

## Supplementary Materials

Supplementary Materials Appendix 1: sensitivity analyses of electroacupuncture vs antidepressants for HAMD-24. Appendix 2: forest diagram of SDS for electroacupuncture vs sham-electroacupuncture. Appendix 3: forest diagram of SDS for electroacupuncture vs antidepressants in follow-up.
