# Supplementary material for: Effectiveness and Safety of Electroacupuncture for Depression: A Systematic Review and Meta-Analysis
Source: Evid Based Complement Alternat Med. 2022 Aug 18;2022:4414113. doi: 10.1155/2022/4414113 (PMC9410808; doi:10.1155/2022/4414113)
Supplement: Supplementary Materials — Appendix 1: sensitivity analyses of electroacupuncture vs antidepressants for HAMD-24. Appendix 2: forest diagram of SDS for electroacupuncture vs sham-electroacupuncture. Appendix 3: forest diagram of SDS for electroacupuncture vs antidepressants in follow-up. [file 4414113.f1.zip › Appendix3 Forest diagram of SDS for electroacupuncture VS antidepressants in follow-up.pdf]

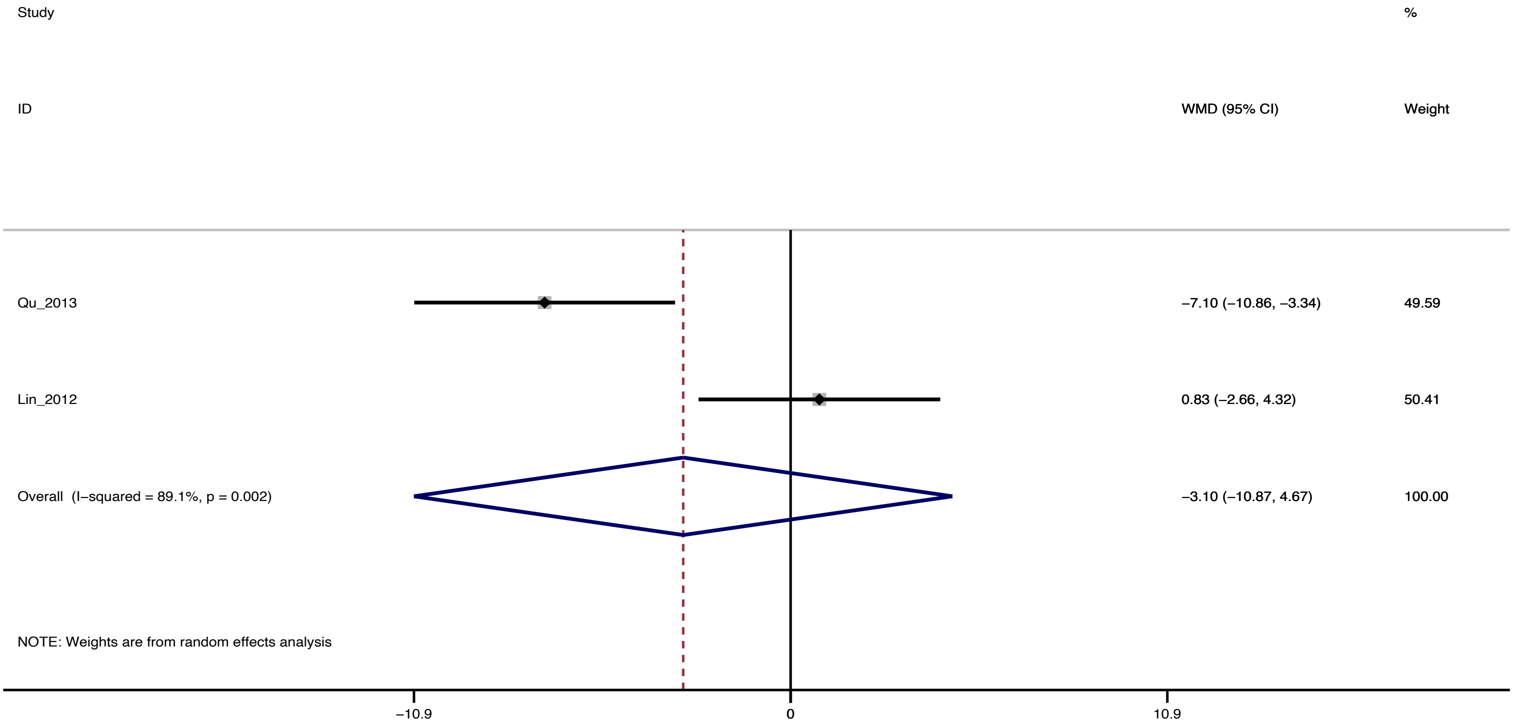
Study
ID
%
Weight
WMD (95% CI)
Qu_2013
−7.10 (−10.86, −3.34)
49.59
Lin_2012
0.83 (−2.66, 4.32)
50.41
Overall (I−squared = 89.1%, p = 0.002)
−3.10 (−10.87, 4.67)
100.00
NOTE: Weights are from random effects analysis
−10.9
0
10.9
