# Supplementary figures and images for: Effectiveness and Safety of Electroacupuncture for Depression: A Systematic Review and Meta-Analysis
Source: Evid Based Complement Alternat Med. 2022 Aug 18;2022:4414113. doi: 10.1155/2022/4414113 (PMC9410808; doi:10.1155/2022/4414113)

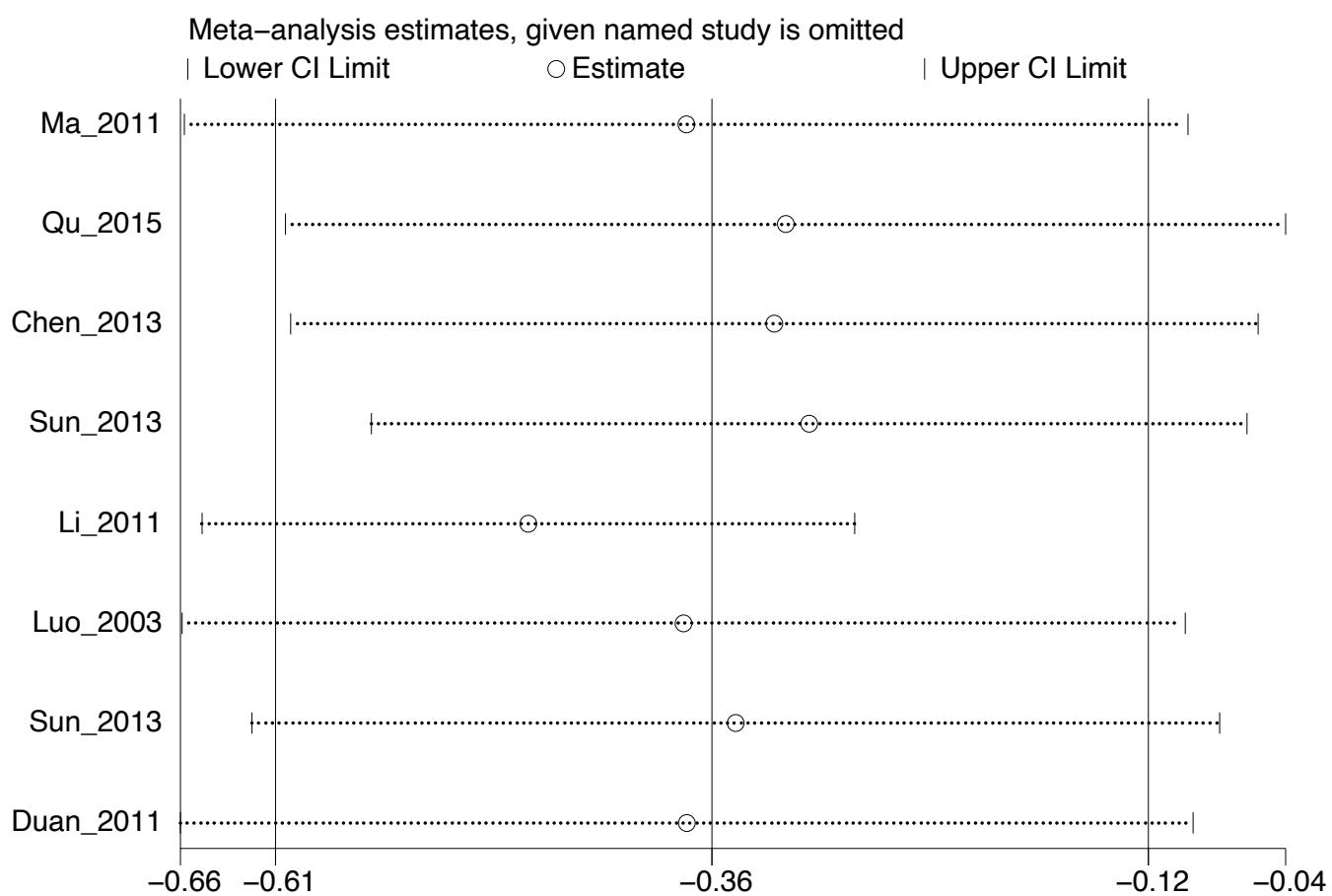

Supplement: Supplementary Materials — Appendix 1: sensitivity analyses of electroacupuncture vs antidepressants for HAMD-24. Appendix 2: forest diagram of SDS for electroacupuncture vs sham-electroacupuncture. Appendix 3: forest diagram of SDS for electroacupuncture vs antidepressants in follow-up. [file 4414113.f1.zip › Appendix1 Sensitivity analyses of electroacupuncture VS antidepressants for HAMD-24.pdf]

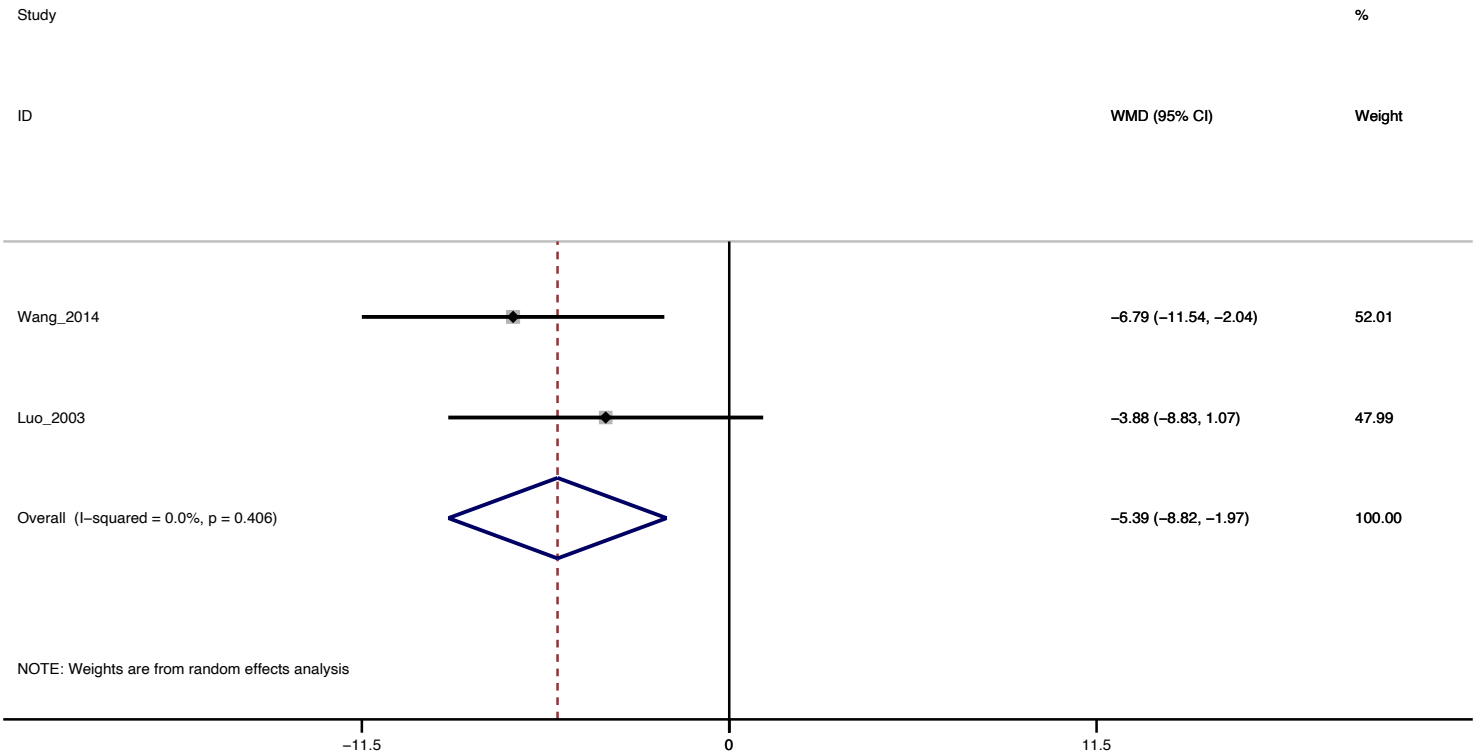

Supplement: Supplementary Materials — Appendix 1: sensitivity analyses of electroacupuncture vs antidepressants for HAMD-24. Appendix 2: forest diagram of SDS for electroacupuncture vs sham-electroacupuncture. Appendix 3: forest diagram of SDS for electroacupuncture vs antidepressants in follow-up. [file 4414113.f1.zip › Appendix2 Forest diagram of SDS for electroacupuncture VS sham-electroacupuncture.pdf]
